# A Transgenic Mouse Model to Track MRC1-High Macrophages Using In Vivo Optical Imaging

**DOI:** 10.3390/ijms27104305

**Published:** 2026-05-12

**Authors:** Chintan Chawda, Giorgia Zambito, Natasa Gaspar, Christopher Schliehe, Pieter J. M. Leenen, Clemens Löwik, Laura Mezzanotte

**Affiliations:** 1 Department of Radiology and Nuclear Medicine, Erasmus MC Cancer Institute, Erasmus University Medical Center Rotterdam, Molenwaterplein 40, 3015 GD Rotterdam, The Netherlands; c.chawda@erasmusmc.nl (C.C.); giorgiazambito87@gmail.com (G.Z.); natasa.gaspar@gmail.com (N.G.); lowik.clemens57@gmail.com (C.L.); 2 Department of Molecular Genetics, Erasmus MC Cancer Institute, Erasmus University Medical Center Rotterdam, 3015 CN Rotterdam, The Netherlands; 3 Department of Immunology, Erasmus MC Cancer Institute, Erasmus University Medical Center Rotterdam, 3015 CN Rotterdam, The Netherlands; c.schliehe@erasmusmc.nl (C.S.); p.leenen@erasmusmc.nl (P.J.M.L.)

**Keywords:** transgenic reporter mice, optical in vivo imaging, macrophages, mannose receptor C-type 1, CD206, pancreatic ductal adenocarcinoma

## Abstract

Macrophages play a crucial role in health and disease. Currently, reporter mice for tracking alternatively activated macrophages in vivo are lacking. We designed a transgenic mouse model in which luminescence and fluorescence proteins, click beetle red luciferase (CBRED2) and mKate2, report on the expression of the Mrc1/Cd206 promoter, active in the monocyte/macrophage population. The mouse line was named B6Mrc1-*mKate2-CBRED2*. Using this novel mouse model, we were able to develop in vitro assays to validate transgenic macrophage polarization and test them with compounds of repolarization potency. Furthermore, in the in vivo assays, we exploited the migratory and infiltrative potency of macrophages for detecting tumor locations via optical imaging. In fact, macrophages can act as universal cancer markers, as they infiltrate primary and secondary tumors, stimulating or suppressing tumor growth. We first characterized transgenic mice for reporter expression ex vivo, followed by the generation of luminescence-based assays to reflect the polarity of differentiated macrophages, and lastly, we visualized reporter macrophages accumulating and infiltrating the tumor microenvironment (TME) of murine pancreatic ductal adenocarcinoma (PDAC) at multiple time points. We found that the extent of macrophage recruitment and retention was dependent on the infiltrative T-cell and dendritic cell populations present in the TME, reflecting the immunologically hot or cold nature of the PDAC clones, respectively. In conclusion, the ability to optically detect light-emitting macrophages can be applied not only for cancer studies but also in the context of inflammatory diseases.

## 1. Introduction

Macrophages are a key component of the cellular immune system. They contribute to the maintenance of tissue homeostasis in conjunction with inducing and regulating immune responses. Additionally, macrophages can contribute to cancer progression, resistance [1,2,3], and suppression [4,5] through diverse mechanisms. They are highly plastic immune cells that have historically been subdivided into two major phenotypes when activated in vitro. On the one hand, pro-inflammatory or classically macrophages are induced after exposure to bacterial components, such as lipopolysaccharides (LPSs), or pro-inflammatory factors, such as interferon (IFN)-γ. On the other hand, alternatively activated macrophages have been characterized as immunosuppressive cells that develop in response to immune-regulatory cytokines such as interleukin (IL)-4 or IL-13 [6,7,8]. In vivo, macrophages with different polarization and activation markers have been shown to coexist in tissues. For example, tumor-associated macrophages (TAMs) have been shown to promote cell proliferation and tissue repair and are defined as M2-like [9,10,11]. TAMs shape an immunosuppressive microenvironment (TME), the tumor stroma, and are associated with tumor progression [12,13]. In line with these observations, the number of TAMs alongside their activation within the TME was described to correlate with unfavorable prognosis [14,15].

MRC1 (mannose receptor C-type 1), also known as CD206, is a type 1 membrane receptor expressed by macrophages and mediates endocytosis of glycoproteins. This receptor protein exhibits high affinity for mannose-rich structures [16], such as those found on the surfaces of bacteria, fungi, and viruses. Their engulfment and disintegration consequently lead to antigen processing and presentation on molecules of the major histocompatibility complex (MHC), which is central for the initiation and regulation of adaptive immunity. In this study, classically activated macrophages with LPS and IFN-γ have been characterized as macrophages with low MRC1 expression levels. In contrast, alternatively activated macrophages with IL-4 and IL-10 were characterized by the upregulation of MRC1 expression [17] and have been described as MRC1-high macrophages [18,19]. Consequently, MRC1 expression has been utilized in several studies as a marker to visualize tumor-promoting macrophages/TAMs [20,21,22,23,24].

Previously, transgenic mouse models using a single reporter molecule, specifically dedicated for imaging of macrophages during inflammation and tissue injury, have been introduced [25]. However, similar models for multimodal imaging of macrophages are still lacking. To bridge this gap, we developed a transgenic knock-in mouse model in which the expression of click beetle red luciferase (CBRED2) and the fluorescent protein mKate2 are driven by the Mrc1 promoter (B6-Mrc1-*mKate2-CBRED2* mice).

In this study, we first demonstrate the application of the transgenic B6-Mrc1-*mKate2-CBRED2* mice in an in vitro setting by distinguishing MRC1-high macrophages from MRC1-low macrophages based on Mrc1 promoter-driven bioluminescence emission. In addition, previous studies have shown that macrophages migrate towards the TME [26] in mammary carcinoma [27], colorectal tumors [28], and melanoma [29]. We built upon this knowledge and adoptively transferred reporter macrophages from transgenic B6-Mrc1-*mKate2-CBRED2* mice into mice bearing murine pancreatic adenocarcinoma. This allowed us to successfully visualize macrophage migration and recruitment dynamics into the pancreatic TME using multimodality optical in vivo imaging. The key contribution of this mouse model is its potential to visualize macrophages non-invasively, with bioluminescence reflecting the viability status of macrophages in real time and fluorescence serving as ex vivo confirmation or an eventual intravital imaging solution.

## 2. Results

### 2.1. Characterization of Transgenic B6-Mrc1-mKate2-CBRED2 Macrophage Reporter Mice

**Figure 1:**
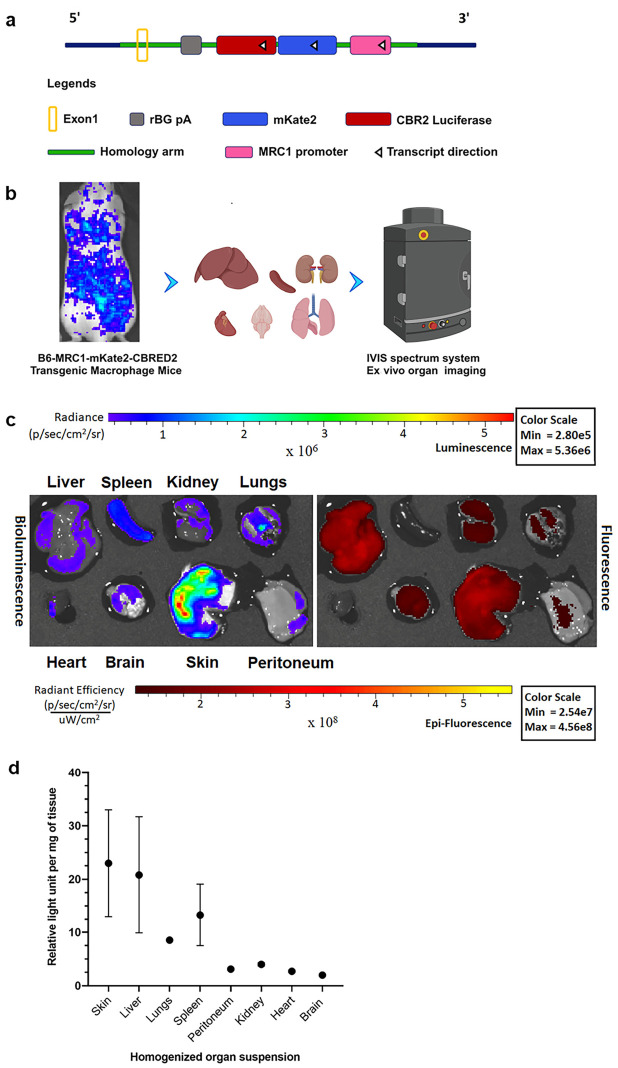
(**a**) Schematic representation of B6-Mrc1-*mKate2-CBRED2* construct used to generate transgenic mice. Exon 1 represents the first exon of the ROSA26 locus, and rBG pA represents the rabbit beta-globin polyadenylation signal. The transcript was flanked by homology arms of the DNA sequence next to the cutting site and was targeted to the mouse ROSA26 locus. (**b**) Drawing of organ-specific luminescence from B6-Mrc1-mKate2-CBRED2 mice. Created in BioRender. Mezzanotte, L. (2026) https://BioRender.com/pvuw2da (accessed on 2 January 2024). (**c**) Representative images of ex vivo organ characterization from B6-Mrc1-mKate2-CBRED2 mice for MRC1 promoter-driven CBRED2 BL emission and mKate2 FL (*n* = 3, biological replicates). For the left BL image, ex vivo CBRED2 luciferase-generated BL emission (radiance) was recorded in the presence of D-luciferin (1 mM) from the given organs. Organs measured are as indicated (from left to right, respectively). Color scale bar, is depicted as radiance (p/sec/cm^2^/sr) as seen above, where the images are within a minimum luminescence of 2.80 × 10^5^ and a maximum of 5.36 × 10^6^ (*n* = 3 mice, biological replicates). Right image: Ex vivo mKate2 protein-generated FL (radiant efficiency) from the given organs. The mKate2 filters in IVIS imaging systems were set at 580 nm for excitation and 640 nm for emission. Color scale values are depicted as radiant efficiency (p/sec/cm^2^/sr)/µw/cm^2^ (*n* = 3 mice, biological replicates). (**d**) In vitro BL emission in terms of relative light unit per milligram of organ from B6-Mrc1-*mKate2-CBRED2* mice. One-way ANOVA was employed for the test, and the graph was generated using Prism Graphpad software version 9 with *n* = 3 mice, biological replicates. Error bars represent ± SD.

The transgenic mouse model was generated using a construct in which a key regulatory element of the Mrc1 promoter drives two optical reporter genes, the one coding for fluorescent protein mKate2 [30,31] fused to the N-terminus of *CBRED2* luciferase gene [32] (Figure 1a). To validate this novel mouse model, we initially performed an ex vivo characterization of organ-specific MRC1 expression (Figure 1b). Indicated organs were dissected, and CBRED2 luciferase-specific bioluminescence (BL) emission as well as fluorescence (FL) signals emitted by the mKate2 reporter were quantified (*n* = 3, biological replicates). CBRED2-specific BL (Figure 1c, left) and mKate2-specific FL (Figure 1c, right) emissions were detected from the liver, spleen, kidneys, lungs, heart, brain, skin, and peritoneum of the reporter mice. Furthermore, BL (Figure 1c, left) was higher in the skin, lungs, and liver compared to other organs. This was also reflected by the FL (Figure 1c, right) except for the spleen, where mKate2 signals were quenched, possibly by hemoglobin FL absorption related to the high amount of blood present in this organ (Figure 1c, right). For other organs, such as the peritoneum, heart, and kidneys, the signal was at the level of background fluorescence (Figure 1c, right), whereas the bioluminescent signal confirmed the Mrc1 promoter activity in these organs (Figure 1c, left). Furthermore, we quantified the BL from 1 milligram of homogenized tissue from the respective organs. We found a 10-fold higher signal emission in homogenates from the liver and skin in comparison to that from the brain and heart. Furthermore, BL emission from the spleen was observed to be 6-fold higher compared to that from the brain. Lastly, the lungs gave a 3-fold higher emission relative to the brain (Figure 1d). These findings align with the reported mouse organ-specific MRC-1 transcript (molar concentration of RNA), which exhibited nearly 3-fold higher levels in adult lungs compared to that observed in the whole brain [33]. We further confirmed the presence of double-positive CD206-mKate2 cells ex vivo in the brain as microglial cells and perivascular macrophages (Supplementary Figure S1a), demonstrating that mKate2 expression in the cytosol is correlated to the presence of CD206 in the membranes of specific cells.

### 2.2. In Vitro Polarization of Bone Marrow-Derived Macrophages

**Figure 2:**
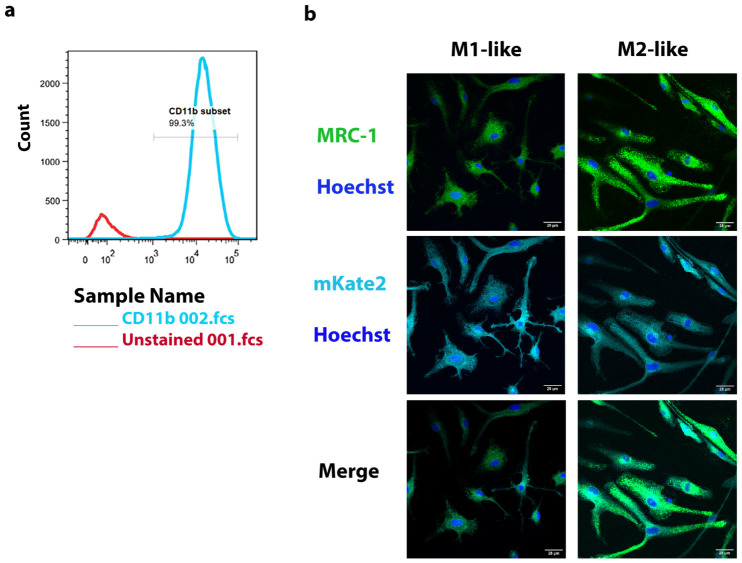
(**a**) Flow cytometry was performed to identify and quantify CD11b-expressing cells from primary bone marrow-derived macrophage cultures. Four days after initial cell culture establishment in the presence of M-CSF, the cells were detached and stained for CD11b. (**b**) Confocal imaging of polarized M1-like and M2-like macrophages. MRC1 protein is visualized by immunofluorescence in green, and FL emission from mKate2 is depicted as cyan. Nuclei stained by Hoechst is seen in blue color.

For the in vitro characterization of transgenic B6-Mrc1-*mKate2-CBRED2* reporter macrophages, we initially isolated and differentiated bone marrow progenitor cells into bone marrow-derived macrophages using murine M-CSF for 7 days. Using fluorescent staining for the surface marker CD11b for macrophages in combination with flow cytometry, we confirmed the myeloid identity of around 99% of the cells based on gating for the CD11b marker (Figure 2a). Next, we were able to morphologically distinguish activated macrophages at 48 h of polarization (Figure 2b). Classically activated macrophages upon treatment with LPSs and IFN-γ demonstrated a flat and round epithelial cell-like structure with spiky protrusions extending from the cell membrane. In contrast, alternatively activated macrophages attained by IL-4 treatments were found to have a fibroblast-like morphology with elongated extensions. Non-stimulated macrophages appeared to be smaller and moderately elongated. When performing immunofluorescence staining, the MRC1 protein and mKate2 were found to be expressed in each type of macrophage but with a relatively higher mean fluorescence intensity in alternatively activated macrophages (Supplementary Figure S1b; Figure 2b).

**Figure 3:**
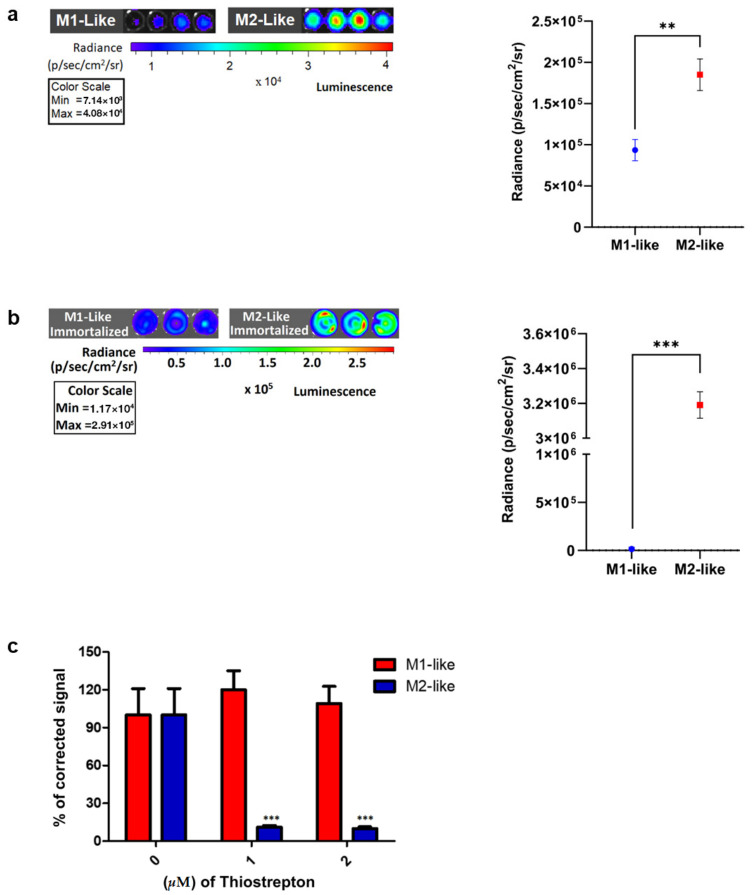
(**a**) Representative in vitro BL emission imaged in black glass-bottom 96-well plates from primary polarized macrophages, polarized for 72 h with LPS+IFN-γ or IL-4, respectively. Cells in the wells were incubated with 1 mM D-luciferin substrate for 10 min in the dark prior to image acquisition. Each condition was performed in four replicates, and experiments were repeated twice. Color scale representation in the wells above denotes MRC1 promoter activity driving reporter *CBRED2* BL gene expression. Color scale bar in radiance (p/sec/cm^2^/sr) (*n* = 4, technical replicates). The graph represents in vitro MRC1 promoter-driven CBRED-2 luciferase luminescence from in vitro classically activated macrophages, as seen in blue, and alternatively macrophages, as seen in red. A paired test was employed, and the graph was generated using Prism Graphpad software version 9. Luminescence readings were measured from 4 wells per condition each of a black glass-bottom 96-well plate: *p* value < 0.01 (**). (**b**) Representative in vitro BL emission imaged in black glass-bottom 96-well plates from immortalized and polarized M1-like and M2-like Hoxb8-derived macrophages, treated for 72 h with LPS+IFN-γ or IL-4, respectively. Cells in the wells were incubated with 1 mM D-luciferin substrate for 10 min in the dark prior to image acquisition. Each condition was performed in three replicates, and experiments were repeated twice. Color scale representation in the wells above denotes MRC1 promoter activity driving reporter CBRED2 BL gene expression. Color scale bar in radiance (p/sec/cm^2^/sr) (*n* = 3, technical replicates). The graph represents in vitro MRC1 promoter-driven CBRED-2 luciferase luminescence from in vitro immortalized and classically activated Hoxb8-derived macrophages, as seen in blue, and alternatively activated Hoxb8-derived macrophages, as seen in red. A paired test was employed, and the graph was generated using Prism Graphpad software version 9. Luminescence readings were measured from 3 wells per condition each of a black glass-bottom 96-well plate; *p* value < 0.001 (***). (**c**) Graph showing the normalized BL signal emitted from polarized primary B6-Mrc1-mKate2-CBRED2 macrophages when treated with increasing concentrations of thiostrepton (*n* = 3, technical replicates). *p* < 0.0001 was calculated by multiple comparisons and one-way ANOVA using Prism Graphpad software version 9. Error bars represent ± SD.

To validate Mrc1 promoter-driven CBRED2 expression, we assayed the luminescence generated by macrophages activated as described before (Figure 3a), considering that the BL emission obtained is, in general, a sensitive readout. These experiments showed that luminescent signals emitted from alternatively activated macrophages were significant, being 2-fold higher in comparison to those from classically activated macrophages (*n* = 4, technical replicates) (Figure 3a), confirming higher Mrc1 promoter activity in alternatively activated macrophages relative to classically activated macrophages. This observation was in line with earlier data presented by Orecchioni M. and colleagues [20], where IL-4 treatment led to increased expression of MRC1 in alternatively activated macrophages. Of note, our results also point out that there is a basal level of MRC1 promoter activity in classically activated macrophages in vitro.

Since, primary macrophages require fresh bone marrow for each differentiation, we immortalized primary bone marrow precursor cells by recombinant expression of estrogen-inducible HoxB8 [29]. In this way, the macrophage reporter system can be used without requiring mice. This allowed us to differentiate HoxB8-derived macrophages (HBDMs) that exhibited similar polarization potency as compared to primary bone marrow-derived macrophages. Also, in using HBDMs, the alternatively activated macrophage population showed higher (approx. 10-fold) bioluminescent signals compared to the same number of classically activated HBDMs, where luminescence was found below the detection level (*n* = 3, technical replicates) (Figure 3b).

To evaluate the potential of reporter macrophages to assay the ability of specific compounds to induce repolarization of macrophages, we treated activated macrophages with thiostrepton and performed BL measurements. Thiostrepton is an antibiotic produced by several species of Streptomyces, and it is an FDA-approved anti-microbial drug. High-throughput phenotypic screening and transcriptional analysis identified new compounds and targets for macrophage reprogramming [34], showing that a concentration of 2 µM thiostrepton could repolarize alternatively activated macrophages into a macrophage population associated with the expression of the pro-inflammatory cytokines tumor necrosis factor-alpha (TNF-α) and IL-1β. Here, we show that 1 µM and 2 µM thiostrepton resulted in a decrease in CBRED2-specific BL emission from alternatively activated macrophages (Figure 3c). When alternatively activated macrophages were treated with thiostrepton, the corrected signal emission was nearly 9 times lower compared to untreated cells, suggesting downregulated activity of the Mrc1 promoter. This effect was specific for alternatively activated macrophages and did not affect MRC1 expression in classically activated macrophages (*n* = 3, technical replicates) (Figure 3c).

### 2.3. Imaging Accumulation of B6-Mrc1-mKate2-CBRED2 Macrophages in Two Different Pancreatic Carcinoma Clones with Distinct Tumor Microenvironments

We next generated reporter macrophages from the bone marrow of B6-Mrc1-mKate2-CBRED2 mice and tested their ability to serve as a tumor biomarker in recipient BL6 mice bearing subcutaneous pancreatic carcinomas. In a proof-of-concept experiment, we investigated the accumulation of injected reporter macrophages within the TME of pancreatic carcinomas. Importantly, we quantified the accumulation of our reporter macrophages within the TME of two different KrasG12D/+; Trp53R172H/+; P48-Cre (KPC) murine pancreatic carcinoma clones in vivo, namely KPC 2838c3 (T-cell high/immune infiltrative/“hot” clone) and KPC 6694c2 (T-cell low/“cold” clone).

**Figure 4:**
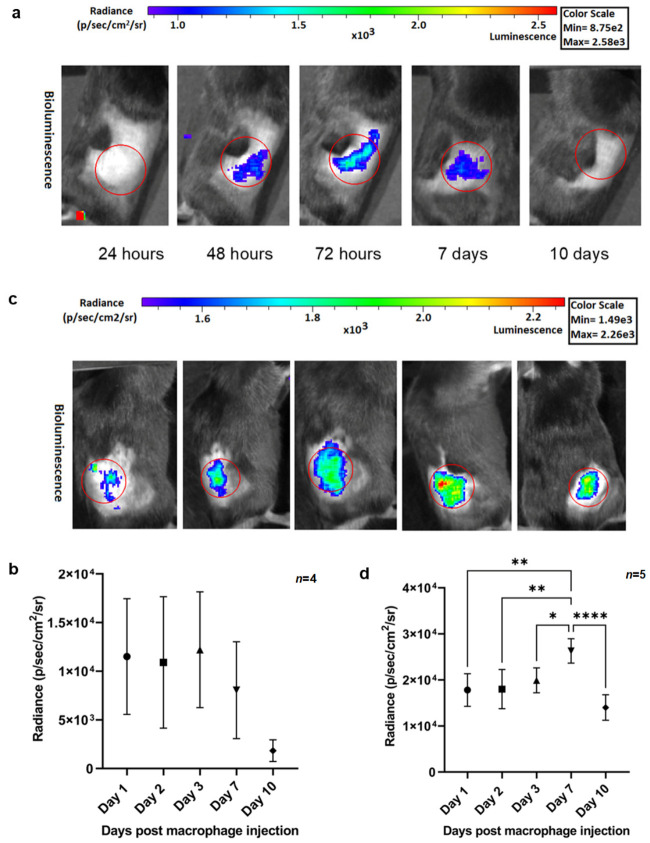
(**a**) In vivo BL imaging of B6-Mrc1-mKate2-CBRED2 transgenic macrophages from the tumors at different time points. Mice were injected subcutaneously with 3 × 10^5^ hot KPC tumor cells and 11 days after implantation, 10 × 10^6^ B6-Mrc1-*mKate2-CBRED2* macrophages were injected intravenously. Images were taken of the same mice on days 1, 2, 3, 7, and 10 post-macrophage injection in hot KPC tumor-bearing mice. Color scale bar indicated above the images. (**b**) Representative in vivo BL imaging of a single B6-Mrc1-*mKate2-CBRED2* transgenic mouse showing signal derived from macrophages from the region of interest (ROI) at and around cold KPC 6694c2 pancreatic tumors at different time points. Mice were injected subcutaneously with 3 × 10^5^ cold KPC tumor cells, and 11 days after implantation, 10 × 10^6^ B6-Mrc1-mKate2-CBRED2 macrophages were injected intravenously. Images were taken of the same mice on days 1, 2, 3, 7, and 10 post-macrophage injection in cold KPC tumor-bearing mice. Color scale bar indicated above the images. (**c**) The graph shows BL radiance recorded from transgenic macrophages accumulating in hot KPC 2838c3 pancreatic tumor-bearing test mice (*n* = 4, biological replicates) (mean photon flux from the region of interest (ROI)). The luminescence readings as a visual reference are represented from a single representative mouse, as shown in (**a**). The images were taken at indicated time points post-B6-Mrc1-mKate2-CBRED2 macrophage injection in KPC 2838c3 hot tumor-bearing C57BL/6 mice. Statistical comparisons were performed using a one-way ANOVA. Error bars represent ± SD. (**d**) The graph shows the quantified and plotted BL radiance recorded from transgenic macrophages accumulating in cold KPC 6694c2 pancreatic tumor-bearing test mice (*n* = 5, biological replicates) (mean photon flux from the region of interest (ROI)). The luminescence readings as a visual reference are represented from a representative mouse, as shown in (**b**). The images were taken at the indicated time points post-B6-Mrc1-mKate2-CBRED2 macrophage injection in KPC 6694c2 cold tumor-bearing C57BL/6 mice. Error bars represent ± SD. Attributed significance denotes *p* < 0.05 (*), *p* < 0.01 (**), and *p* < 0.0001 (****).

Specifically, the hot carcinoma clone KPC 2838c3 is characterized by higher infiltration of intratumoral CD3^+^ T cells, CD3^+^CD4^+^ T-helper cells, and CD3^+^CD8^+^ cytotoxic T cells in vivo [35], in comparison to the cold carcinoma clone KPC 6694c2. Additionally, there is an increased presence of CD103^+^ dendritic cells and higher CD3^+^CD8^+^ cytotoxic T-cell infiltration with lower granulocytic myeloid-derived suppressor cells (gMDSCs) present in the tumor stroma of the hot KPC 2838c3 clone [36]. Considering the nature of this clone, we questioned whether the related environment would recruit less macrophages compared to the cold carcinoma clone KPC 6694c2, induce their polarization, or allow for shorter retention and survival of our injected reporter macrophages in its tumor stroma. To validate this, longitudinal BL imaging for B6-Mrc1-mKate2-CBRED2 macrophages introduced in mice harboring the hot KPC 2838c3-derived tumors was conducted. During dorsal imaging of hot KPC 2838c3 tumor-bearing mice, BL signals emitted from reporter macrophages in the tumor stroma of a single representative mouse are seen in Figure 4a, and quantitative analysis of luminescence from reporter macrophages calculated from all mice shows the signal reaching peak emission at day 3 (Figure 4b). We additionally saw a temporal increase in signal-based accumulation of reporter macrophages days 2 and 3 in the lungs (Supplementary Figure S2a,b) (*n* = 4, biological replicates). Lastly, following 10 days of the in vivo BL imaging session, we conducted an ex vivo organ analysis for BL emissions from organs of C57BL/6 mice bearing hot KPC 2838c3 carcinomas that received the transgenic macrophages. BL emission was detected from surviving B6-Mrc1-*mKate2CBRED2* macrophages present in the liver (Supplementary Figure S3a). Together, with the in vivo and ex vivo BL image analysis, we demonstrated the retention and survival of injected reporter macrophages peaking from day 2 till day 3 within the TME of the hot KPC 2838c3 tumor stroma. In analogy, we administered bone marrow-derived B6-Mrc1-*mKate2-CBRED2* macrophages intravenously into C57BL/6 mice (10 × 10^6^ cells per mouse) bearing subcutaneous cold KPC 6694c2 carcinomas. BL imaging of mice with administered with CBRED2-expressing reporter macrophages showed an accumulation of cells in the TME from day 1 till day 10 (Figure 4c) (image of single mouse for visual representation) and 4d (*n* = 5, biological replicates). Furthermore, the quantitative analysis displayed a temporal increase in BL emission emitted from CBRED2-expressing reporter macrophages on day 7, reflecting transgenic macrophage accumulation in the TME (Figure 4d). BL imaging showed an accumulation and retention of transgenic macrophages in and around the lungs, due to entrapment originating from bolus injection of a large amounts of cells and most likely due to the size of the pulmonary capillary (5–10 µm) in comparison to the size of macrophages (10–20 µm) (Supplementary Figure S2c,d). BL imaging for organs of C57BL/6 mice with cold KPC tumors revealed that after injection, reporter macrophages were found to be localized mostly in the liver, as evident from BL (Supplementary Figure S3b) emission measurements. Furthermore, ex vivo visualization of our exogenous reporter macrophages at the cellular level within the cold pancreatic tumor microenvironment was conducted. Confocal imaging exhibited co-localization of immuno-stained MRC1and mKate2 proteins originating from transgenic reporter macrophages (Supplementary Figure S3c row 1 (R1), images 1 and 2) on the same slide. Near the vasculature, transgenic reporter macrophages expressing mKate2 FL (588 nm) are shown as magenta, and immunostaining for MRC1 is shown co-localized in green, as expected (Supplementary Figure S3c, row 2 (R2), images 1 and 2). BL signals originating from macrophages in cold tumors were seen peaking by 3-fold for day 7 and nearly 2-fold for day 10 in comparison to the signals generated from hot tumors for the same time (Supplementary Figure S3d): we speculate that this may reflect the tendency of macrophages to accumulate more in cold tumors or that the Mrc1 promoter is highly induced in the cold TME. In summary, B6-Mrc1-mKate2-CBRED2 reporter macrophages were successful in accumulating in the TMEs of both hot KPC 2838c3 and cold KPC 6694c2 tumors, which could be visualized non-invasively over a prolonged period.

## 3. Discussion

In this study, we introduce B6-Mrc1-mKate2-CBRED2 mice and confirm the applicability of the optical transgene to reflect the Mrc1 promoter activity in differentiated transgenic macrophages in vitro and ex vivo. In addition to this, we detected bioluminescent macrophages in vivo by using an adoptive immune cell transfer system over a longitudinal period.

In vitro, bone marrow-derived myeloid progenitor cells isolated from the B6-Mrc1-mKate2-CBRED2 mouse could be differentiated into macrophages. Furthermore, these newly differentiated macrophages were polarized by classical or alternative factors towards two distinct phenotypes. Thiostrepton, an FDA-approved antibiotic drug and a translation blocker [37], was tested as a pro-inflammatory macrophage-promoting/polarizing compound [34]. A decrease in light emission of alternatively activated macrophages suggested repolarization of macrophages to a pro-inflammatory phenotype. Based on this validation, we could envision that primary and immortalized HoxB8-derived macrophages derived from our new transgenic line may act as a screening platform for macrophage-repolarizing compounds.

Unlike the GFP, EGFP, mCherry, or CFP FL reporters used in other transgenic mouse models [38,39,40,41], we chose the mKate2 transgene. This bright red-emitting FL protein as a reporter opens the possibility of combining our transgenic macrophages with other immune or tumor cell-expressing FL proteins of distinct wavelengths for dual-color in vivo FL intravital imaging studies. We anticipate that the distinctiveness of the mKate2 FL protein will be valuable for future applications in dual-color FL imaging studies using our B6-Mrc1-*mKate2-CBRED2* model and intravital microscopy. Our initial characterization of the transgenic line using the mKate reporter for whole-body in vivo imaging presented suboptimal results due to high background fluorescence, making it difficult to detect mKate2 FL emitted from organ-specific macrophages present in the liver, lungs, or spleen. B6-Mrc1-*mKate2-CBRED2* transgenic reporter mice represent a complementary addition to a set of previously developed macrophage reporter mice [38,39,40,41]. A recently generated model for in vivo macrophage tracking is the LysM-LG reporter mouse [38]. In brief, a lysozyme-M promoter-driven Cre recombinase reflected the state of the macrophages. RLuc luciferase-generated BL reflected a basal state, while upon Cre-mediated RLuc excision, expression of CBRED2 BL-reflects activated macrophages state. In comparison, LysM-LG reporter mice in vivo reported a basal or activated state of the macrophages, while transgenic reporter macrophages from B6-Mrc1-mKate2-CBRED2 mice successfully reflected the polarity of alternatively activated macrophages. Although the dichotomy M1/M2 to describe the functional state of macrophages in vivo has been updated [42], complementing CD206-MRC-1 tracking with a CD163 promoter as a driver for reporter genes will be an additional tool to track the M2-like activation state of macrophages during in vivo imaging.

Furthermore, during the adoptive transfer of our reporter macrophages, we assessed two murine pancreatic carcinoma clones. These clones were used to examine the difference in recruitment and survival of our reporter macrophages. These clones were initially distinguished based on the ratio of CD103^+^ DCs and CD8^+^ T-cell infiltration to gMDSCs in the tumor stroma [36]. The “cold” KPC 6694c2 clone exhibited lower T-cell infiltration compared to the “hot” KPC 2838c3 PDAC carcinoma clone [36]. From our in vivo findings, we infer that the cold KPC tumor stroma allows for a prolonged retention of reporter macrophages in comparison to the hot KPC tumor stroma. We speculate that the cold KPC tumor clone, characterized by increased CXCL1 production along with a higher number of dendritic cells and MDSCs, contributed to macrophage survival over time [36]. We hypothesize that this retention may be attributed to differences in the profile of chemokines or survival factors produced in the TME, which promotes survival in “cold” KPC tumors or increases the activity of MRC-1 promoters. Although it is possible that reporter expression is increased due to the tumor microenvironment, we observed a higher signal from the beginning of the experiment, suggesting increased recruitment in cold tumors. S100 calcium-binding protein A9 (S100A9) is one of such proteins that promotes an M2-like polarization state in the PDAC stroma and positively correlates with M2-like macrophage marker CD206. It is found to be secreted by stromal monocytes [43] and enhanced by pancreatic stellate cells [44] when macrophages were exposed to PDAC cell-derived exosomes containing S100A9 [45]. Interestingly, when considering the intravenous (tail vein) administration of macrophages and the route of pulmonary circulation, we observed a similar trend in macrophage survival and signal emission from the lungs of mice with the cold KPC 6694c2 clone compared to the hot KPC 2838c3 clone. Our findings agreed with the observations of Zhu Y and colleagues [9], where circulating monocytes were found to accumulate in the TME of pancreatic carcinoma.

## 4. Materials and Methods

### 4.1. Generation of B6-Mrc1-mKate2-CBRED2 Reporter Macrophage Mice

The generation of the reporter plasmid and breeding of B6-Mrc1-mKate2-CBRED2 macrophage reporter mice were performed in collaboration with Taconic Inc. The mice were generated by co-injecting a single guide RNA, Cas9 mRNA, and the donor vector cassette containing “rBG pA-KozakMRC1 promoter-mKate2CBRED2” into fertile albino C57BL/6 mice eggs. *mKate2* gene was fused to click beetle luciferase (*CBRED2*) gene at the N-terminus. The single guide RNA was complementary to the mouse ROSA26 locus. This was aimed at generating promoter-dependent knock-in insertion within offspring. Following the sequence analysis of PCR-amplified DNA, the founder F0 pups were identified, isolated, and further crossed with wild-type mice to establish a B6-Mrc1-mKate2-CBRED2 F1 generation. All animal experiments were approved by the ethical committee of the Erasmus University Medical Center, Rotterdam, The Netherlands. Animal care and handling was performed according to the regulations and guidelines provided by the Dutch Experiments on Animal Act (WoD) and the European Directive for the protection of animals used for scientific purposes (2010/63/EU) (project license: 17-867-81, study plan ID: SP2100045, number: 88). The albino variant of C57BL/6 mice was purchased from Charles River Laboratory (Leiden, The Netherlands). B6-Mrc1-mKate2-CBRED2 transgenic reporter mice and C57BL/6 mice of 6–10 weeks of age were placed at three–four mice per cage, where male and female mice were separately placed and provided access to food and water ad libitum and were hosted in the animal facility at the Erasmus MC, Rotterdam, The Netherlands.

### 4.2. Generation of Primary Bone Marrow-Derived Macrophages (BMDMs)

Primary bone marrow-derived macrophages (BMDMs) were generated and cultured as described in ref. [46] with modifications. Animals were sacrificed by cervical dislocation under isoflurane anesthesia. During the collection of bone marrow cells, the bones were submerged in 3 mL of DMEM containing 10% FBS and 1% penicillin–streptomycin and then crushed and ground in a sterile mortar under aseptic conditions. The bone marrow cell suspension was then filtered through a 100-micron cell strainer and cultured in DMEM with 10% FBS and 1% penicillin–streptomycin (final concentration of 100 I.U./mL penicillin and 100 μg/mL streptomycin) supplemented with recombinant murine macrophage colony-stimulating factor (M-CSF) factor (50 ng/mL) (Peprotech, Thermo Fisher, Whaltam, MA, USA, #315-02) on non-cell culture-coated plates. After 72 h, the free-floating suspension cells/debris in the media were washed off and renewed with fresh media with M-CSF for the differentiating macrophages attached to the surface. To detach the macrophages at 80% confluency (day 7), 2 mM EDTA in PBS was used and incubated at 37 °C and 5% CO_2_ for 10 min. These cells were then considered non-polarized M0 macrophages.

### 4.3. Generation of Immortalized HoxB8-Derived Macrophages (HBDMs)

HoxB8-immortalized bone marrow precursor cells were generated as described previously [29]. Animals were sacrificed by cervical dislocation under isoflurane anesthesia. Briefly, single cell suspensions of C57BL/6J-derived bone marrow (prepared as described above for BMDMs) were cultured in cytokine-rich RPMI medium containing 10% FBS, 1% PS, 10 ng/mL recombinant mouse interleukin 3 (IL-3, BioLegend San Diego, CA, USA, #575502, San Diego, CA, USA), 10 ng/mL recombinant mouse interleukin 6 (IL-6, BioLegend San Diego, CA, USA, #575702), and 50 ng/mL recombinant mouse stem cell factor (SCF, BioLegend San Diego, CA, USA, #579702) for 3 days to induce the proliferation of precursor cells. Next, cells were immortalized by retroviral transduction of the estrogen-inducible HoxB8 vector pMSCV_Hoxb8_ER, using the packaging vector pCL-Eco for virus production (both vectors originally contributed by F. Schmitz). On the day after transduction, the cytokine-rich medium was replaced with HoxB8 medium (complete RPMI with 10 ng/mL granulocyte macrophage colony-stimulating factor (GM-CSF, PeproTech #315-03) and 1 μM β-estradiol (β-EST, Sigma-Aldrich, Merck, Darmstad, Germany, #E2758)). Suspension cells were passaged every 2–3 days and maintained for 2 weeks to select immortalized precursors. For differentiation into HBDMs, cells were collected and washed 2 times with PBS to remove β-EST, and the medium was changed to standard BMDM medium, as described above. The differentiation of HBDMs was then performed as described above for BMDMs and cells were harvested on day 7 for experiments.

### 4.4. Polarization of BMDMs

Classically activated macrophages were generated by polarizing macrophages for 72 h in presence of *E. coli* strain O111:B4 lipopolysaccharide (LPS) (Sigma-Aldrich, # L2630) (100 ng/mL) and IFN-γ (10 ng/mL) (Peprotech, Cranbury, NJ, USA, #31505) in DMEM supplemented with M-CSF (50 ng/mL), 10% FBS, and 1% penicillin–streptomycin. Similarly, for alternatively activated macrophages, polarizing factors were M-CSF (50 ng/mL) and murine IL-4 (10 ng/mL) (Sigma-Aldrich, #SRP3211). Macrophages were exposed to the supplementary factors in the media for 72 h to observe polarized phenotypes.

### 4.5. Immunostaining and Immunocytochemistry

At day four post-polarization, both macrophage types were fixed using 4% PFA for 10 min at room temperature in PBS, and staining was performed against MRC1/CD206 using rat anti-mouse CD206 monoclonal antibody (1:100) (Bio-Rad, Hercules, CA, USA, #MCA2235T). As a secondary antibody, Alexa fluor 488 donkey anti-rat Ig (1:500) (Invitrogen, Thermo-Fisher, Whaltam, MA, USA, # A-21208) was used.

Tumor tissue was isolated from the test mice (KPC tumor-bearing C57BL/6 mice injected with B6-Mrc1-mKate2-CBRED2 macrophages) and control mice (KPC tumor-bearing C57BL/6 mice) at days 3 and 10 post-transgenic macrophage administration. Tumor tissues were flash frozen in liquid nitrogen and sectioned with a width of 10 microns using a Cryotome cryostat from LABEC. Tumor sections were then fixed using 4% PFA in PBS. Sections were permeabilized using 5% FBS/0.05% Tween in PBS followed by immunostaining using rat anti-mouse CD206 monoclonal antibody (1:200) with the secondary antibody Alexa fluor 488 donkey anti-rat Ig (1:500). Next, rabbit polyclonal antibody against mkate2 (1:1000) (Anti-tRFP antibody, evrogen, Moscow, Russia, #AB233) was applied, followed by the secondary antibody Alexa fluor 647 donkey anti-rabbit Ig (1:1000). Nuclei in cells and tissue sections were stained using Hoechst 33342 (1:1000) (ThermoFisher, #62249) for 10 min, washed, and lastly mounted with Aquamount (BDH, London, UK, #362262H). The confocal images were acquired using a Zeiss Near-infrared Wide-Field microscope and were analyzed in FIJI Image J software, version (1.54r).

### 4.6. Ex Vivo and In Vitro BL Imaging

Animals were sacrificed by cervical dislocation under isoflurane anesthesia. Ex vivo characterization of organs from B6-Mrc1-mKate2-CBRED2 mice involved the analysis of MRC1 expression in the liver, lungs, spleen, kidneys, brain, heart, skin, and peritoneal tissues, and the samples were imaged for BL emission. Out of 24 mice from which bone marrow was extracted to establish a monocyte-derived macrophage culture, we utilized organs from three mice to conduct this ex vivo characterization study. First, 1 mM D-luciferin solution prepared in sterile water was pipetted onto the organs followed by incubation for 10 min at room temperature. The tissues were then analyzed in the IVIS Spectrum imager (Revvity, Whaltam, MA, USA) for BL (open filter). For in vitro characterization of the organs from B6-Mrc1-mKate2-CBRED2 reporter mice, 100 mg of each of the above-mentioned tissues was homogenized in PBS using TissueLyser LT (Qiagen, Hilden, Germany) and resuspended in the provided buffer from the ONE-Glo™ Luciferase Assay System (Promega, Madison, WI, USA, #E6110), followed by taking the readings in the IVIS Spectrum imaging system. In the case of KPC tumor-bearing C57BL/6 mice with injected exogenous B6-Mrc1-mKate2-CBRED2 macrophages, similar steps for ex vivo organ-specific BL characterization were performed. Here, selected organs (liver, lungs, spleen, and tumor) were incubated for 10 min with D-luciferin and imaged in the IVIS Spectrum imager for BL. In vitro MRC1 promoter-driven BL emission for classically and alternatively activated macrophages was measured as follows: First, 1.5 × 104 naïve macrophages were seeded per well in Biolite-96 optical-bottom black cell culture plates (Thermo scientific, # 130336) and polarized as previously mentioned. On day 4, cells were supplemented with 1 mM D-luciferin per well and placed in a 37 °C incubator at 5% CO_2_ for 10 min followed by imaging in the IVIS Spectrum imager for BL, as described above. Thiostrepton (Merck, 598226-1GM) was used as a macrophage-repolarizing agent in an in vitro setting. B6-MRC1-CBRED2-mKate2 reporter macrophages were plated at a density of 4 × 10^4^ cell per well of glass-bottom 96-well plates and polarized accordingly with factors to obtain activated macrophages. Next, increasing concentrations of thiostrepton (1 µM and 2 µM) were supplemented in triplicate wells for each macrophage subtype and incubated for 24 h. Finally, cells were incubated with 1 mM D-luciferin and MRC1-specific BL was measured in an IVIS Spectrum in vivo imaging system (Revvity, Waltham, MA, USA) for BL as described above. Data were normalized for the number of viable cells in the wells.

### 4.7. In Vivo Experiments and Whole-Body Bioluminescence Imaging

C57BL/6 mice were injected subcutaneously in the flanks with 3 × 10^5^ KPC cells from the clones 2838c3 (hot) or 6694c2 (cold), with 6 mice each, respectively, as previously described [23]. To calculate the number of animals required for each experimental group with significant differences in light output, we used the statistical formula N = 2 (Zα/2 + Zβ) 2 (SD/D) 2. The formula number required was 6 (N = 5.8, biological replicates). Following 10 days of subcutaneous tumor growth, 2838c3 (hot) or 6694c2 (cold) tumor-bearing C57BL/6 mice were injected separately with 10 × 10^6^ B6-Mrc1-mKate2-CBRED2 macrophages intravenously via tail vein injection. Two mice from each group of hot and cold tumors were not injected with transgenic macrophages and were designated as control mice. The selection of control mice was conducted by simple randomization via a lottery method where the mouse IDs were randomly selected to designate mice in the control group. These mice were imaged and used to calculate background emission. Tumor clones were kindly provided by Prof. B. Stanger, UPENN. For whole-body in vivo imaging, the ophthalmic ointment Puralube^®^ (Dechra, Northwich, UK) was applied to the eyes of mice to prevent corneal damage and drying of the eyes during prolonged inhalation anesthesia by isoflurane USP in the IVIS Spectrum in vivo imaging system (Revvity, Waltham, MA, USA). For BL measurements, a standard dose of D-luciferin (150 mg/kg) was injected intraperitoneally. Then 10 min later, BL image acquisition was performed in the IVIS scanner under anesthesia with settings of open filter for auto and 30 s exposure using FOV C and medium binning. Imaging of mice was performed under isoflurane (2%).

For signal quantification, identical ROIs were placed on the region where signal emission was maximal, while the background signal was subtracted using the ROI value obtained from non-luminescent/fluorescent regions.

### 4.8. Image Analysis and Statistics from In Vivo Imaging Sessions

BL image data from in vivo whole-body and ex vivo organs were analyzed in Living Image Software (Revvity) version 4.8.4. Statistical analysis was performed using one-way ANOVA for repeated measurement and a subsequent Tukey’s post-test for multiple comparisons, along with graphical representations performed in the software Graphpad Prism 7. No inclusion or exclusion criteria were established during the image analysis. Results were reported as the mean ± STDEV or SEM, and significance was attributed when *p* < 0.05 (*), *p* < 0.01 (**), *p* < 0.001 (***), or *p* < 0.0001 (****), as described in figure legends. To reduce researcher bias, blinding was ensured. The technician injecting the mice with tumor cells was aware of group allocation at different stages of our in vivo studies.

### 4.9. Animal Welfare

The humane end point included a sudden loss in body weight, the tumor burden reaching the maximum (1.5 cm^3^), or any illness being observed.

## 5. Conclusions

We successfully established a novel B6-Mrc1-*mKate2-CBRED2* transgenic reporter mouse line. The isolated B6-Mrc1-*mKate2-CBRED2* macrophages from our newly generated transgenic reporter mouse line could be used for developing (imaging-based) assays for in vitro screening of macrophage-polarizing, -suppressing, or -reprogramming drugs. B6-Mrc1-*mKate2-CBRED2* macrophages, when administered intravenously, undergo dynamic redistribution in vivo and preferentially accumulate in the tumor site over time, with a distinct accumulation trend observed across hot and cold PDAC models.

## Data Availability

The main data are displayed in the manuscript and Supplementary Material. The raw data are made available on the data repository Zenodo, link: https://zenodo.org/records/20111297 (accessed on 14 April 2026).

## Supplementary Materials

The following supporting information can be downloaded at: https://www.mdpi.com/article/10.3390/ijms27104305/s1.

## Abbreviations

CBRED2	click beetle red luciferase
TME	tumor microenvironment
PDAC	pancreatic ductal adenocarcinoma
LPSs	lipopolysaccharides
IFN	interferon
IL	interleukin
TAMs	tumor-associated macrophages
MRC1	mannose receptor C-type 1
MHC	major histocompatibility complex
BL	bioluminescence
FL	fluorescence
M-CSF	macrophage colony-stimulating factor
HBDMs	HoxB8-derived macrophages
TNF-α	tumor necrosis factor alpha
KPC	KrasG12D/+; Trp53R172H/+; P48-Cre
gMDSCs	granulocytic myeloid-derived suppressor cells
