# Supplementary material for: A Transgenic Mouse Model to Track MRC1-High Macrophages Using In Vivo Optical Imaging
Source: Int J Mol Sci. 2026 May 12;27(10):4305. doi: 10.3390/ijms27104305 (PMC13207300; doi:10.3390/ijms27104305)
Supplement: Supplementary file 1 [file ijms-27-04305-s001.zip › ijms-4199365-supplementary.pdf]

S1a

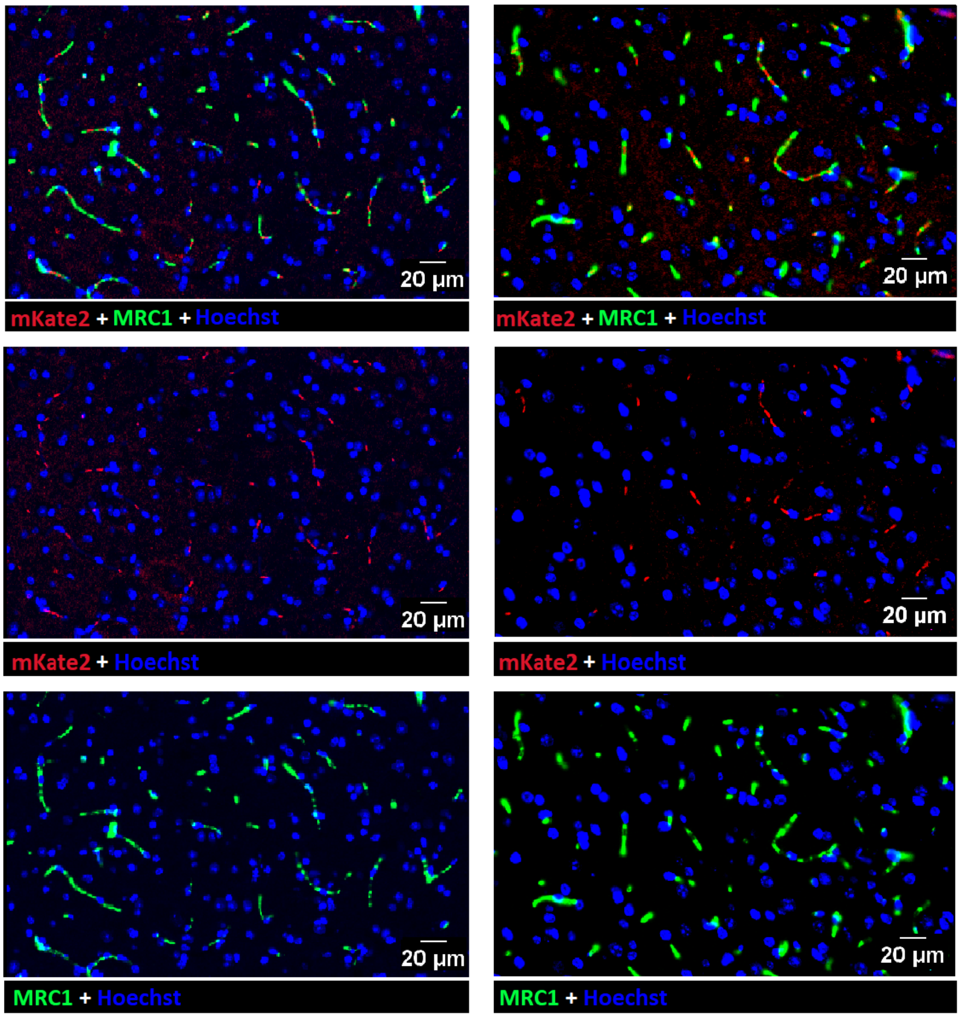

Supplementary Figure S1a: Immunocytochemistry of the brain tissue section from B6-Mrc1-mKate2-CBRED2 transgenic macrophage reporter mice. Image represents the merged images of green (MRC1 protein), red (mKate2 FL protein) and blue FL depicts the nucleus of the cells using Hoechst 33342. The immunostaining was performed against MRC1/CD206 using CD206 monoclonal antibody complemented with secondary antibody Alexa fluor 574.

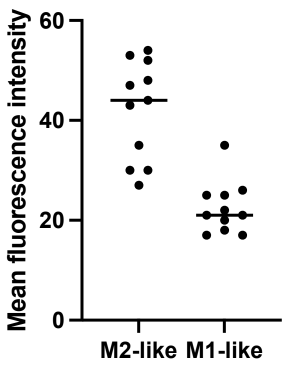

Supplementary Figure S1b: quantification of mean fluorescence intensity per cell in M2-like versus M1-like mKate2 expressing isolated macrophages stained for MRC-1.

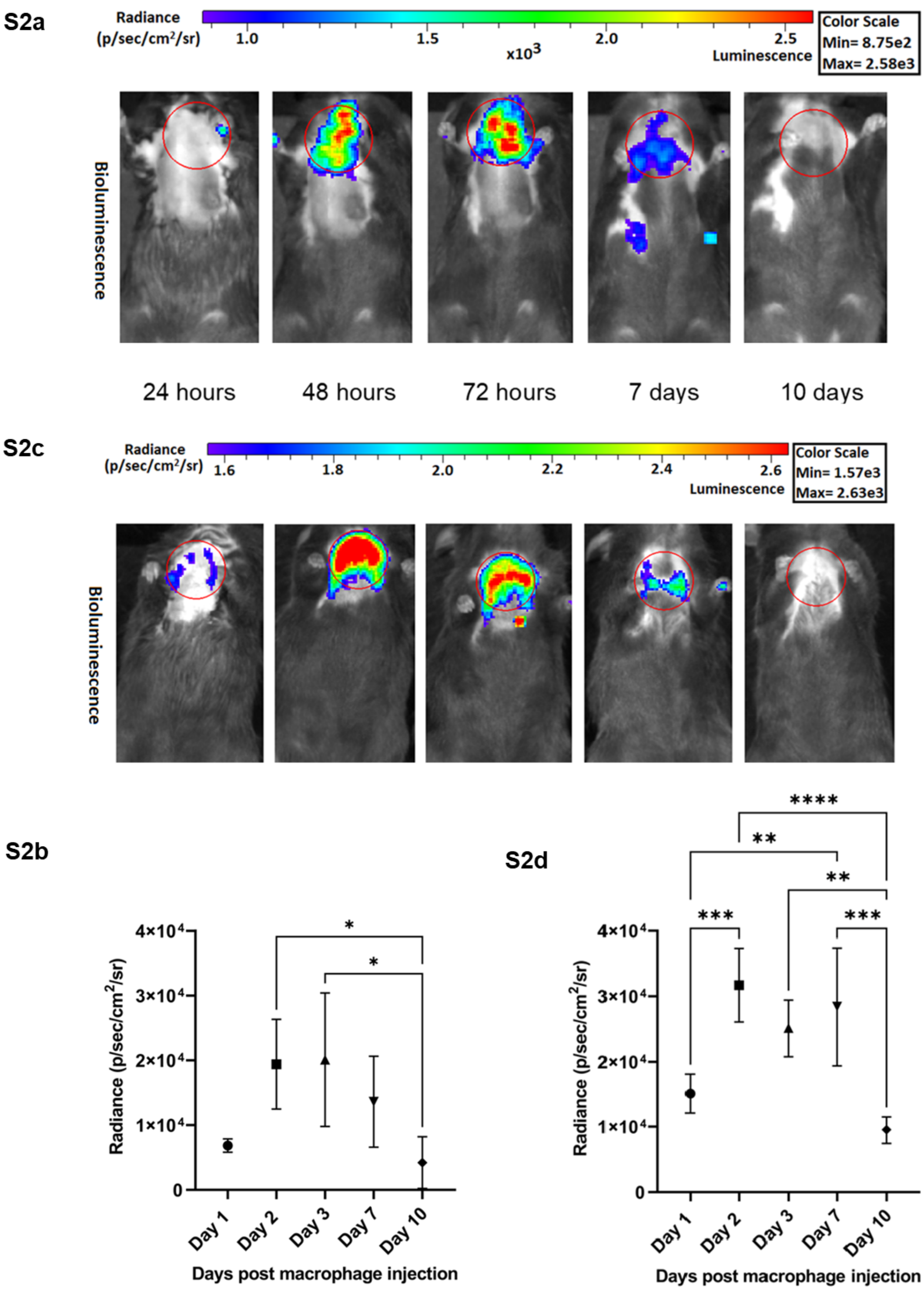

Supplementary Figure S2: a) *In vivo* BL imaging of B6-Mrc1-mKate2-CBRED2 transgenic macrophages from the region of interest (ROI) at and around lungs in a single representative hot KPC 2838c3 pancreatic tumor-bearing mice at different time points. Mice were injected subcutaneously with $3x10^5$ hot KPC tumor cells and 11 days after implantation, $10x10^6$ B6-Mrc1-mKate2-CBRED2 macrophages were injected intravenously. Images were taken of the same mice on days 1, 2, 3, 7 and, 10 post macrophage injection in hot KPC tumor bearing mice. Color scale bar indicated above the images. b) The graph shows the BL radiance recorded for the all the test mice (n=5 mice) imaged facing the ventral side, (mean photon flux from the region of interest (ROI)). The luminescence readings as a visual reference is represented from a

single mice as shown in Supplementary Figure 2a. The images were taken at indicated time points post B6-Mrc1-mKate2-CBRED2 macrophage injection in KPC 2838c3 hot tumor bearing C57BL/6 mice. Statistical comparisons were performed using a one-way ANOVA. Error bars represent ±STDEV. Attributed significance denotes $p < 0.05$ (*). c) Representative *in vivo* BL imaging of B6-Mrc1-mKate2-CBRED2 transgenic macrophages from the region of interest (ROI) at and around lungs of a single representative cold KPC 6694c2 pancreatic tumor-bearing mice at different time points. Mice were injected subcutaneously with $3x10^5$ cold KPC tumor cells and 11 days after implantation, $10x10^6$ B6-Mrc1-mKate2-CBRED2 macrophages were injected intravenously. Images were taken of the same mice on days 1, 2, 3, 7 and, 10 post macrophage injection in cold KPC tumor bearing mice. Color scale bar indicated above the images. d) The graph shows the BL radiance recorded for the all the test mice (n=5 mice) imaged facing the ventral side, (mean photon flux from the region of interest (ROI)). The luminescence readings as a visual reference is represented from a single mice as shown in Supplementary Figure 2d. The images were taken at indicated time points post B6-Mrc1-mKate2-CBRED2 macrophage injection in cold KPC 6694c2 tumor bearing C57BL/6 mice. Statistical comparisons were performed using a one-way ANOVA. Error bars represent ±STDEV. Attributed significance denotes $p < 0.05$ (*), $p < 0.01$ (**), $p < 0.001$ (***) and $p < 0.0001$ (****).

S3a

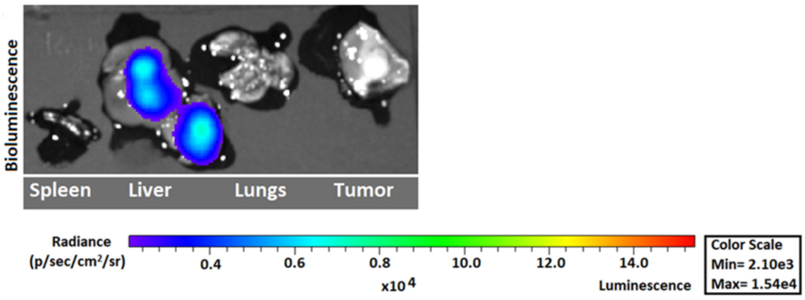

S3b

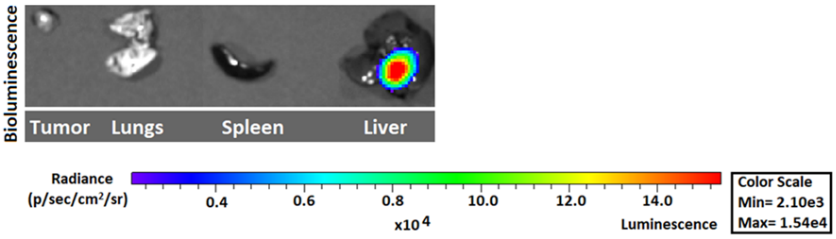

S3c

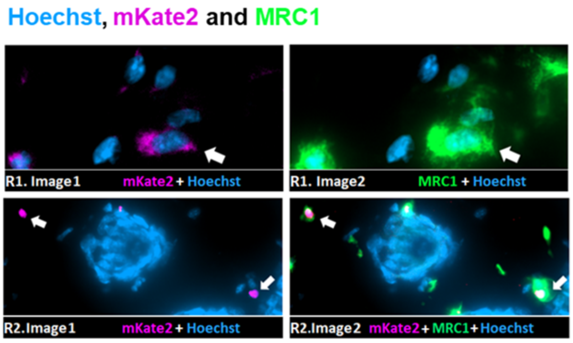

S3d

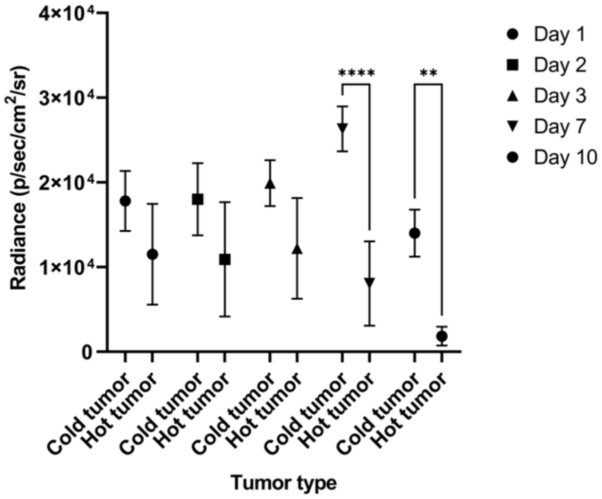

Supplementary Figure S3: a) *Ex vivo* BL emission images of spleen, liver, lungs and tumor (from left to right) from hot KPC 2838c3 pancreatic tumor bearing mice. C57BL/6 mice were euthanized, and organs were isolated on day 10 after B6-MRC1-CBRED2-mKate2 macrophages were injected into tumor bearing C57BL/6 mice. 200 µl of 1 mM D-luciferin in PBS were pipetted on each organ mentioned above, incubated in dark for 10 minutes at room temperature prior to imaging using the IVIS spectrum systems. Scale bar demonstrating the BL emission range is present as a reference above the image. b) *Ex vivo* BL emission images of tumor, lungs, spleen and liver (from left to right) from cold KPC 6649c2 pancreatic tumor bearing mice. C57BL/6 mice were euthanized, and organs were isolated at day 3 after B6-MRC1-

CBRED2-mKate2 macrophages injected in tumor bearing C57BL/6 mice. 200 µl of 1 mM D-luciferin in PBS was pipetted on each organ mentioned above, incubated in dark for 10 minutes at room temperature prior to imaging in IVIS spectrum systems. Scale bar demonstrating the BL emission range is present as a reference above the image. c) Immunocytochemistry of tumor cryosection in which B6MRC1-CBRED2-mKate2 macrophage infiltrating cold 6694c2 KPC pancreatic tumors at day 3 post macrophage administration is shown. The first row (R1) shows two images: Image 1 exhibits B6-MRC1-CBRED2mKate2 macrophage where magenta represents mKate2 fluorescent protein. The mKate2 protein was stained using rabbit polyclonal Ig complemented with secondary antibody Alexa fluor 647 donkey anti-rabbit Ig. Image 2 displays similar B6-MRC1-CBRED2-mKate2 macrophage present in the first image where green depicts MRC1 protein expressed on macrophages. The immunostaining was performed against MRC1/CD206 using rat anti-mouse CD206 monoclonal antibody complemented with secondary antibody Alexa fluor 488 donkey anti-rat. Images in the second row (R2) shows two images: Image 1 displays mKate2 protein from B6-MRC1-CBRED2-mKate2 macrophages. Here, magenta represents mKate2-specific fluoresce. The mKate2 protein was stained using rabbit polyclonal Ig complemented with a secondary antibody Alexa fluor 647 donkey anti-rabbit Ig. Image 2 represents the merged images of green (MRC1 protein), magenta (mKate2 FL protein) from image 1 and 2 of column 2. In all the images of column 1 and 2, blue FL depicts the nucleus of the cells using Hoechst 33342. d) The graph compares the BL radiance recorded for the all the test mice (n=5 for each cold and hot tumor bearing mice) implanted with cold 6694c2 KPC vs hot 2838c3 KPC tumor imaged at each time points facing the ventral side, (mean photon flux from the region of interest (ROI)). Statistical comparisons were performed using a one-way ANOVA. Error bars represent ±STDEV. Attributed significance denotes $p < 0.05$ (*)
